# The use of meta-analyses for benefit/risk re-evaluations of hydroxyethyl starch

**DOI:** 10.1186/s13054-015-0940-7

**Published:** 2015-06-02

**Authors:** Christian J Wiedermann

**Affiliations:** Department of Internal Medicine, Central Hospital of Bolzano Bozen, Teaching Hospital of the Medical University of Innsbruck, Lorenz-Böhler Street 5, 39100 Bolzano, Bozen Italy; Interdisciplinary Medical Research Center South Tyrol (IMREST), Lorenz-Böhler Street 5, 39100 Bolzano, Bozen Italy

Whether hydroxyethyl starch (HES) is safe for perioperative use is not known. Evaluating HES in cardiac surgery, Jacob and colleagues suggested conclusion was of a more favorable safety profile for HES 130/0.4 [1]. The authors, however, wrongly stated that they followed the Preferred Reporting Items for Systematic reviews and Meta-Analyses (PRISMA) guidelines because the meta-analysis lacked a table of included study characteristics, an assessment of bias, identification of any pre-specified subgroup analyses, and tests of interaction to compare different HES solutions. In many included studies, the control group received HES, patients with heavy bleeding were excluded *post hoc*, and anti-fibrinolytic drugs were used to minimize bleeding. For instance, in two included trials the group assigned to gelatin actually received significantly more postoperative HES 130/0.4 than the group allocated to HES 130/0.4 [2,3]. In an included trial of HES 130/0.4, the results from one of the four study centers were excluded *post hoc* without explanation [4]. Other sources of bias were aggregation of intra- with postoperative blood loss and preferential use of calculated rather than measured blood loss. Furthermore, no tests of interaction were presented even to address the question of whether HES 130/0.4 differs from other HES solutions in its effect on bleeding risk after cardiac surgery.

Between-study heterogeneity was shown to considerably affect the use of meta-analyses for drug-safety alert issues [5]. Even though meta-analyses may help predict iatrogenic risks, in the field of perioperative volume resuscitation with HES quality of meta-analytic evidence is still too poor to reliably inform drug-regulatory authorities. This technique should not replace further assessments during benefit/risk ratio re-evaluations of HES for perioperative use.

## Abbreviation

HES: Hydroxyethyl starch
